# Targeting transforming growth factor‐β signalling for cancer prevention and intervention: Recent advances in developing small molecules of natural origin

**DOI:** 10.1002/ctm2.795

**Published:** 2022-04-05

**Authors:** Devesh Tewari, Anu Priya, Anusha Bishayee, Anupam Bishayee

**Affiliations:** ^1^ Department of Pharmacognosy School of Pharmaceutical Sciences Lovely Professional University Phagwara Punjab India; ^2^ Department of Pharmacology School of Pharmaceutical Sciences Lovely Professional University Phagwara Punjab India; ^3^ Pine View School Osprey Florida USA; ^4^ College of Osteopathic Medicine Lake Erie College of Osteopathic Medicine Bradenton Florida USA

**Keywords:** apoptosis, cancer, in vitro, in vivo, natural products, proliferation, TGF‐β signalling, treatment

## Abstract

**Background:**

Cancer is the world's second leading cause of death, but a significant advancement in cancer treatment has been achieved within the last few decades. However, major adverse effects and drug resistance associated with standard chemotherapy have led towards targeted treatment options.

**Objectives:**

Transforming growth factor‐β (TGF‐β) signaling plays a key role in cell proliferation, differentiation, morphogenesis, regeneration, and tissue homeostasis. The prime objective of this review is to decipher the role of TGF‐β in oncogenesis and to evaluate the potential of various natural and synthetic agents to target this dysregulated pathway to confer cancer preventive and anticancer therapeutic effects.

**Methods:**

Various authentic and scholarly databases were explored to search and obtain primary literature for this study. The Preferred Reporting Items for Systematic Reviews and Meta‐Analysis (PRISMA) criteria was followed for the review.

**Results:**

Here we provide a comprehensive and critical review of recent advances on our understanding of the effect of various bioactive natural molecules on the TGF‐β signaling pathway to evaluate their full potential for cancer prevention and therapy.

**Conclusion:**

Based on emerging evidence as presented in this work, TGF‐β‐targeting bioactive compounds from natural sources can serve as potential therapeutic agents for prevention and treatment of various human malignancies.

## Graphical abstract

- TGF‐β is a superfamily of evolutionarily conserved and growth‐inhibitory cytokines that control pleiotropic cellular functions.
- TGF‐β signalling plays a key role in cell proliferation, differentiation, morphogenesis, regeneration and tissue homeostasis implicated in cancer initiation and progression.
- Crosstalk of TGF‐β signalling with other pathways is comprehensively discussed.
- Various bioactive natural compounds modulate the TGF‐β signalling pathway to confer potential cancer preventive and therapeutic activities.

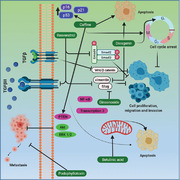

## 1 INTRODUCTION

Hippocrates, the father of modern medicine, coined the word ‘cancer’ and utilised the Greek terms ‘carcinoma’ and ‘karakinos’ to describe tumours.^1^ Cancer is a global health problem, frequently observed in both developed and developing nations; however, lately, there has been a remarkable improvement in survival rates of patients due to early detection and improvement in the development of treatment options.^2^ Cancer represents a hyperproliferative disease characterised by the transformation of cells, apoptotic dysregulation, proliferation, angiogenesis, invasion and metastasis.^3^

Cancer remains a prime reason for mortality, being the second leading cause of death worldwide. About 19.3 million new cases of cancer and around 10.0 million cancer‐related deaths were expected to occur worldwide in 2020.^4^ It has been estimated that there were roughly 1 898 160 cases of cancer diagnosis and approximately 608 570 deaths from cancer in 2021 in the United States,^5^ which is significantly higher than the earlier prediction of the number of expected cases of cancer in the previous year.^6^ Several factors can be attributed to cancer, including physical carcinogens (ultraviolet radiation), chemical carcinogens (asbestos, tobacco and arsenic), and any type of infection from viruses, bacteria, fungi or other parasites. Some other factors include dietary and behavioural problems, low intake of fruits and vegetables, lack of physical exercise and excessive tobacco and alcohol usage.^7^

Treatment of cancer has always been a significant challenge, as there is no complete resolution developed yet for cancer treatment, although its risk can be reduced by avoiding exposure to carcinogens. Although chemotherapy has been standardised and is commonly used for cancer treatment along with surgery, radiation therapy and immunotherapy, often these chemotherapeutic agents can lead to unhealthy levels of toxicity.^8^ Natural products have been an excellent source for drug discovery,^9^, ^10^ as they have more ability than bacteria, fungi, insects and climate to produce structurally diverse primary and specialised compounds.^11^ Natural products are the reservoir of phytoconstituents possessing substantial chemoprotective and anticancer properties and are components of over 60% of currently available anticancer drugs.^12^ It is also noteworthy that ‘combination chemotherapy’, which is widely useful for effective cancer treatment, utilises both natural and synthetic agents.^9^ Several potent plant‐based chemotherapeutic agents such as vinca alkaloids, taxols, epothilones and podophyllotoxins are used commercially.^13^, ^14^ Additionally, there are many different compounds, including curcumin, resveratrol, epigallocatechin gallate (EGCG), genistein, quercetin, lycopene, apigenin, gallic acid, sulforaphane and ursolic acid, which exhibit significant therapeutic effects against cancer.^8^, ^16^, ^17^, ^18^, ^19^, ^20^, ^21^, ^22^, ^23^, ^24^, ^25^

Various phytoconstituents have gained special attention and provided a novel avenue for cancer treatment through targeting different cross‐linked pathways, such as oxidative stress, mutagenesis, inflammation, apoptosis and autophagy, as well as several signalling intermediaries, including cancer metabolism.^26^, ^27^, ^28^ Out of the various other pathways, some prominent therapeutic targets for plant secondary metabolites are the mammalian target of rapamycin (mTOR), phosphatidylinositol 3‐kinases (PI3K), protein kinase B (or Akt), hypoxia‐inducible factor‐1α (HIF‐1α) and extracellular signal‐regulated kinase (ERK),^29^ nuclear factor‐κB (NF‐κB), activator protein 1,^30^ c‐Jun NH_2_‐terminal kinases (JNKs), mitogen‐activated protein kinase (MAPK), tumour necrosis factor (TNF) receptor‐associated factor (TRAF), TNF receptor 1‐associated death domain protein, Janus kinase (JAK) and the signal transducer and activator of transcription (STAT).^26^, ^27^, ^28^, ^29^, ^30^, ^31^, ^32^, ^33^, ^34^, ^35^

The transforming growth factor‐β (TGF‐β) signalling pathway plays a crucial role in various cellular responses; misregulation of this pathway often leads to tumour progression.^36^ The regulatory cytokine TGF‐β has tumour‐suppressive effects, which can be evaded by cancer cells through malignant evolution. TGF‐β can modify various processes, for example, immune regulation, cell invasion and alteration of microenvironments, which may be exploited by cancer cells for their own advantage.^37^

TGF‐β is involved in various biological functions, including embryonic stem cell differentiation and self‐renewal, homeostasis differentiation in cells, immune suppression and the promotion of cancer development.^38^ The characterisation of the TGF‐β signalling pathway is well‐defined. The cell membrane is the location where TGF‐β binds to its receptor and thus initiates a signalling cascade by phosphorylating small mothers against decapentaplegic homolog 2 and 3 (Smad2/3). Afterwards, the phosphorylated Smad2/3 attaches to Smad4, and translocation of the cytoplasmic complex occurs towards the nucleus for activation of transcription of end effectors, including p21, p15 and the parathyroid hormone‐related protein.^39^, ^40^

Several prior reviews are available on different features of TGF‐β signalling. In this line, a review by Lee et al.,^41^ published in 2013, was based on the pros and cons of affecting the TGF‐β pathway during the progression and development of cancer by various natural products. This work was mainly focused on the alteration of gene‐specific DNA methylation levels by natural products in different target tissues and systemic circulations. Another review by Markov et al.^42^ focused on natural triterpenoids and their semi‐ and synthetic‐derivatives on various tumour‐related signalling pathways, including TGF‐β and human epidermal growth factor receptor. A third review published by Farooqi et al.^43^ analysed the role of single phytoconstituent EGCG on the regulation of various deregulated signalling pathways, including TGF‐β, in cancer.

Despite the aforementioned reviews,^41^, ^42^, ^43^ an updated and comprehensive evaluation of the information is still lacking due to the effect of the broader categories of natural products on TGF‐β signalling pathways during tumourigenesis. The available information is limited to drawing conclusive insight into the role of natural and synthetic agents targeting TGF‐β in cancer therapeutics. Therefore, we aim to provide for the first time a comprehensive and critical review of the natural products as well as the synthetic agents that act on TGF‐β along with their mechanistic attributes. The prime objective of this review is to decipher the role of TGF‐β in oncogenesis and to evaluate the potential of various natural and synthetic agents to target this dysregulated pathway to confer cancer preventive and anticancer therapeutic effects. In addition, the review provides insight into the omics approaches associated with TGF‐β in oncology as well as comprehensive preclinical and clinical studies pertaining to various natural products against different cancers.

## 2 TGF‐β RECEPTORS AND SIGNALLING PATHWAY

TGF‐β is a superfamily of evolutionarily conserved and growth‐inhibitory cytokines that control pleiotropic cellular functions.^44^ In normal and primary cancer cells, TGF‐β suppresses the tumour by inducing apoptosis over proliferation.^39^ However, it also encourages tumour metastasis via epithelial‐mesenchymal transition (EMT) stimulation, invasion, migration, chemoattraction and cell adhesion.^45^

### 2.1 TGF‐ß receptors

There are three receptor types, namely, Type Ⅰ (TβRI), Type Ⅱ (TβRII) and Type III (TβRIII), along with seven human type Ⅰ receptors, and five Type Ⅱ receptors; Type III is the most abundant type. Different members of the TGF‐β family bind to a combination of the Types Ⅰ, Ⅱ and III receptors. The TGF‐β isoforms, that is, TGF‐β1, TGF‐β2 and TGF‐β3, usually bind to the Type Ⅱ receptor. Their role is crucial, as they exert both promoter and terminator properties. In normal cells, they indicate tumour‐suppressive features, and in malignant cells, they display tumour‐promoting roles.^46^

TGF‐β Type III is the most abundant type of TGF‐β ligand that is transferred to the signalling receptors TβRI and TβRII, which are occupied with serine/threonine protein kinases at their intracellular domains.^47^ Upon binding of TGF‐β ligand to TβRIII, it either binds to TβRII straight away or presents TGF‐β to TβRII.^48^ Once the TGF‐β ligand binds to the TβRII receptor, it is auto‐phosphorylated and constitutively transphosphorylases the TβRI GS domain, thus stimulating the activity of protein kinase. Activated receptors work with adaptors or cofactors to induce transcriptional activity. TGF‐β through the non‐Smad pathway binds with different cytokines, such as JNK, p38, ERK1/2 and PI3K, which promotes transcriptional activity.^49^

Canonical TGF‐β signalling takes place upon binding of one of the three ligands to TGFBR2, which phosphorylates TGFBR1. Then, this TGFBR1 phosphorylates downstream Smad2 and Smad3 on the carboxy terminus (pSmad2/3C) of a serine residue. Subsequently, it recruits Smad4 and translocates into the nucleus where it controls the transcription of TGF‐β target genes. Upon binding of one of three ligands to TGFBR2, it phosphorylates and recruits TGFBR1, resulting in canonical TGF signalling. Consequently, the downstream Smad2 and Smad3 are phosphorylated by the action of TGFBR1 on a serine residue at its pSmad2/3C (carboxy terminus), which attracts Smad4 and translocates towards the nucleus, where it modulates the transcription of TGF‐targeted genes.^46^

A chimeric receptor model was used to gather more insight. TGFBRs are continuously recycled in the absenteeism of ligands, and ligand interaction only causes heteromeric receptors (TGFBR1/TGFBR2) to be degraded, while homomeric receptors (TGFBR1/TGFBR1) are recycled again to the plasma membrane.^50^

### 2.2 Secretion of TGF‐β

Several cells, including macrophages, secrete TGF‐β. It is secreted either through cancer cells or via cells present on the local stroma, and the attribution of TGF‐β‐induced anticancer T‐cell immunity inhibition can be an adverse event for the host.^51^ Serum proteinases, for instance, plasmin, accelerate TGF‐β release from the complex. This process takes place on the macrophages (surface) in which the latent TGF‐β complex is bound to CD36 by its ligand, thrombospondin‐1. The active TGF‐β release is enhanced by inflammatory stimuli, which are responsible for the activation of macrophages via plasmin activation.^52^ The synthesis of TGF‐β ligands takes place as precursor proteins with a longer N‐terminal pro‐peptide and a shorter mature C‐terminal polypeptide.^53^

### 2.3 Biology and functions of TGF‐β

TGF‐β displays various biological activities, such as chemotaxis, extracellular matrix synthesis, cell differentiation and angiogenesis. It also exhibits growth inhibition of various cells, including endothelial, epithelial and lymphocytic cells. It induces the proliferation of mesenchymal cells, such as fibroblasts. Additionally, it has also been associated with several pathophysiological processes comprising tissue fibrosis, wound repair, morphogenesis and immunosuppression. These bioactivities are usually carried out through specific cell surface receptors, TβR‐I and TβR‐II.^54^, ^55^ TGF‐β plays dualistic roles in tumourigenesis.^56^ In the initial stages, it inhibits cellular growth by blocking tumourigenesis and acts as a tumour suppressor through the regulation of EMT and cell migration. It usually inhibits cell division via cell cycle arrest at the G1 phase. This further enhances the cyclin‐dependent kinase (CDK) inhibitors expression, namely, p15 and p21, with successive c‐Myc suppression, which is a multifunctional oncogene and considered to be present in various human cancers. When it functions as a tumour promoter, it overexpresses itself and leads to invasiveness, cell proliferation and enriched metastatic potential. This is due to regulation in the levels of EMT, angiogenesis and immunosuppression.^57^

TGF‐β dysfunctions lead to diseases such as cancer, connective disorders and fibrotic diseases.^58^ The development of TGF‐β chimaera has emerged recently as a potential approach due to the presence of high‐affinity TGF‐β ligands for their receptors through rearranging, mixing and mutating their binding epitopes.^58^, ^59^

### 2.4 Regulation of TGF‐β

**FIGURE 1:**
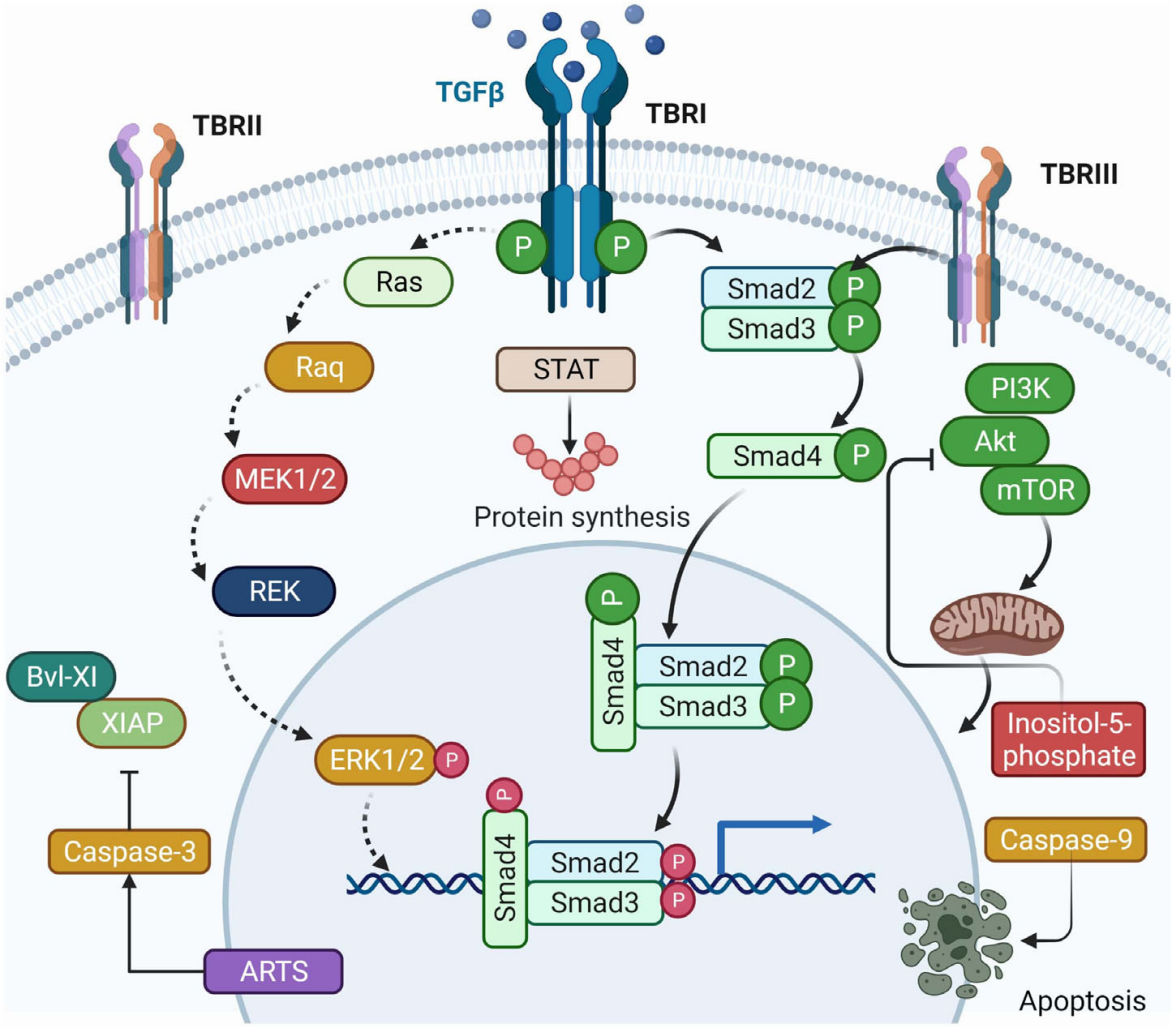
Figure showing molecular pathways involving the transforming growth factor‐β (TGF‐β) role in cancer. The TGF‐β signalling pathway regulates embryogenesis, cell homeostasis, proliferation, differentiation and death. TβRI, TβRII and TβRIII—TGF‐β receptor types—serine/threonine kinases induce heterotetrameric receptor complexes. TGF‐β can carry out apoptosis through small mothers against decapentaplegic (Smad)‐dependent and ‐independent pathways. TGF‐β also follows a non‐Smad pathway where it binds with different cytokine‐like p38, c‐Jun N‐terminal kinases, extracellular signal‐regulated kinase and phosphoinositide 3‐kinase and promotes transcriptional activity. In the nucleus, it regulates gene expression by binding with two types of receptors in the cell membrane with intrinsic serine/threonine kinase activity which is then conducted by intercellular Smad proteins. These are transcription factor that regulates gene expression. Created with BioRender.com

The TGF‐β signalling pathway is largely involved in the cellular process. They have been observed to possess both positive and negative effects by several mechanisms. TGF‐β plays an influential, multifunctional role in cell differentiation and proliferation; however, at different stages, there is a different response of TGF‐β within production, secretion, activation and signalling. In the nucleus, it regulates gene expression by binding with two types of receptors in the cell membrane with intrinsic serine/threonine kinase activity, which is then conducted by intercellular Smad proteins. These are transcription factors that regulate gene expression^60^ as shown in Figure 1. The R‐Smad/Co‐Smad complex attaches with other nuclear cofactor proteins in the nucleus, and the process of gene transcription starts.^60^, ^61^ In the epithelial cells, TGF‐β is believed to function as a regulatory tumour suppressor factor of utmost importance due to its effect of early inhibition of proliferation and apoptosis induction.^62^

## 3 ROLE OF TGF‐β IN TUMOURIGENESIS

### 3.1 Proliferation

TGF‐β is quite efficient in suppressing the proliferative activity of cancer cells in multiple ways. As normal epithelial cells and haematopoietic cells multiply by targeting CDKS, TGF‐β either inhibits CDKS directly by suppressing further proliferation of cells, or it upregulates expression of CDKS inhibitors, such as p15^ink4b^, p27^kip1^ and p21^cip1^. This process inhibits the CDKS complex and causes G1 stage cell cycle arrest. TGF‐β can even inhibit CDK4, which is needed for the progression from the G1 stage to S phase of the cell cycle. Directly inhibiting the cell cycle, it even suppresses the activity of c‐Myc, which inhibits the action of CDKS inhibitors and leads to a reduction in cell proliferation.^49^

TGF‐β, or the TGF‐β/Smad4 signalling pathway in particular, regulates the process of signal transduction from cell membrane to the nucleus. it is also accountable for a broad array of cellular processes, such as differentiation, proliferation, migration and apoptosis. In addition, it is also responsible for the onset and progression of cancer.^63^ As mentioned previously, TGF‐β protein plays a dual role in tumourigenesis. During the initial stages of tumourigenesis, TGF‐β plays a suppressive role. Afterwards, with the tumour progression, cancer cells slowly become resistant, and finally the TGF‐β protein augments immunosuppression of the tumour and facilitates invasion, tumour angiogenesis and metastasis.^64^

### 3.2 Differentiation

TGF‐β expresses its differentiating property differently depending upon the stage of cancer, type of tumour and changes in the tumour microenvironment. During the initial cancer stages, TGF‐β plays a tumour‐suppressive role by arresting the cell cycle and stimulating apoptosis. It can display this activity through the generation of CDK inhibitor p21^Waf1/Cip1^, which is considered the controller of the cell cycle. Cyclin A is a substitute for CDK, and upregulation of cyclin A accelerates the entry of the S phase into NIH3T3 and U937 cells. The complex of cyclin A/CDK is involved in the regulation of progression through the S phase, and it pushes the cell to the G2 phase. Thus, decreasing the expression of cyclin A can stop the cell cycle at the S phase.^65^ TGF‐β has been reported to regulate the immunological response and differentiation of T and B lymphocytes, which are associated with the inflammatory cascade in cancer progression at organ and tissue levels.^66^

### 3.3 Apoptosis

TGF‐β can carry out apoptosis through Smad‐dependent and Smad‐independent pathways. Through Smad‐dependent pathways, TGF‐β is involved in the release of pro‐apoptotic proteins, mediated by TGF‐β‐inducible early response genes, which lead to cellular oxidative stress.^67^ It also activates the death‐associated protein kinase,^68^ which causes apoptosis through mitochondrial cytochrome c release.^68^, ^69^ through inositol‐5‐phosphatase, it downregulates the pro‐survival PI3K‐Akt pathway, which leads to cell death easily.^69^ Through the Smad4‐signalling pathway, the stress‐activated protein kinase/JNK pathway is regulated, which controls the expression of pro‐apoptotic genes, including the Bcl‐2 modifying factor, Bcl‐2 interacting mediator of cell death, Bcl‐2‐associated X protein and caspase‐9. However, cells resistant to TGF‐β for growth inhibition can still react to apoptosis by translocating the mitochondrial apoptosis‐related protein in the TGF‐β signalling pathway to the nucleus, which stimulates caspase‐3 activation and downregulation of Bcl‐xL and X‐linked inhibitor of apoptosis protein expression, leading to apoptosis.^49^, ^69^, ^70^, ^71^, ^72^

### 3.4 Migration

TGF‐β increases the migratory property of cancer cells by re‐constructing the cytoskeleton structure of cells through releasing focal adhesion kinase signalling, stress fibre formation and smooth muscle actin expression.^49^ The changes associated with EMT allow the cells to lose connection with the epithelial cells and easily migrate the cell from the initial position as well as promote cancer metastasis. Like in prostate cancer, by cytoskeleton rearrangement of Cdc42 and RhoA in a dependent fashion and via increasing the stress fibres and lamellipodia,^73^ it promotes the migratory property of cancer cells.^49^, ^74^, ^75^

### 3.5 Cancer initiation and progression

TGF‐β plays a vital dual function in tumour progression by suppressing and promoting it accordingly. The downregulation of TGF‐β is considered vital for the onset of cancer. When cell proliferation is inhibited due to factors, such as CDK inhibitors or by the secretion of anti‐angiogenic factors and through repressing the expression of c‐Myc, it acts as a tumour suppressor. It plays a role as a tumour promoter by activating matrix deposition and agitating immune function, thus stimulating EMT. EMT is an important factor in cancer progression and is described as suppressed epithelial markers and enhanced mesenchymal markers. TGF‐β not only regulates EMT but is also involved in the metastasis of cancer cells. It is considered as a tumour suppressor in malignant cells; however, it acts as a promoter in the metastasis of cancer cells.^76^ TGF‐β’s involvement in cancer microenvironment and its dual roles have been extensively reviewed elsewhere.^66^, ^77^, ^78^, ^79^

### 3.6 TGF‐β downregulation and reactive oxygen species (ROS) p**roductio**n

ROS and TGF‐β both affect cancer progression and tumourigenesis; hence, their mutual effects are very complex. Different cells and tissues produce high amount of ROS through various mechanisms; an increased amount of ROS is a characteristic of all types of cancer as it induces tumour initiation, progression and acts as a secondary messenger for regulating different pathways, such as cell proliferation, survival and apoptosis.^80^ ROS can lead to cancer cells’ apoptosis at the molecular level and cause damage to the nucleic acids.^74^, ^81^

## 4 CROSSTALK OF TGF‐β WITH OTHER PATHWAYS

The TGF‐β is a superfamily of proteins consisting of TGF‐β1, TGF‐β2 and TGF‐β3 isoforms; they are similar in structure but quite distinct in exerting biological response after interacting with different receptors.^82^ Along with TGF‐β, other pathways have roles pertaining to cancer, and they interact differently with receptors and cytokines to produce responses accordingly. Some of the prominent pathways that have interactions with TGF‐ β are presented in this section.

### 4.1 ERK pathway

MAPK is a superfamily of specific protein kinases that transforms extracellular signals to the nucleus and regulates gene transcription; it has an impact largely on the complex cellular process, comprising cell differentiation, proliferation, transformation and apoptosis. MAPK is constituted of subfamilies, such as ERK, JNK and p38/MAPKs. These subfamilies are associated with protein phosphorylation that modifies the cell behaviour and have an ‘on‐and‐off’ mechanism, which is regulated for the treatment of cancer. However, their activation can be triggered by various extracellular molecules, including TGF‐β cytokines.^83^

ERK is one of the major pathways under the MAPK pathway. This pathway is mainly involved in numerous cellular processes, including cell proliferation, differentiation, apoptosis, cell growth and survival. These extracellular signals are conducted and end with the activation of the phosphorylation of proteins. This pathway is activated by RAS/RAF/MEK, which is usually initiated with tyrosine kinase. Even TGF‐β may facilitate the activation of ERK rapidly in cancer cells and normal epithelial cells.^84^ In vitro studies showed that the ERK pathway activation was induced by TGF‐β.^85^

### 4.2 p38 pathway

p38, a stress‐induced kinase activated through several stress stimuli, is involved in various cellular processes. However, p38 can be stimulated by TGF‐β through TAK1 activation and TβR I‐TRAF6 interaction. TGF‐β‐induced p38 activation can augment Smad4 sumoylation via the PIAS family of E3 ligase, hence enabling Smad4‐dependent transcription. Both p38 and TGF‐β activation is vital for the activation of transcription of Agc expression.^86^, ^87^

Interestingly, a crosstalk of TGF‐β, RAC1 and RAC1b on the tumour cells of the breast and pancreas has been demonstrated.^88^ TGF‐β is considered a paradox as depending upon the cancer stage, it can act as both an enhancer or a suppressor of cancer cells to invade, migrate and metastasise through the Smad pathway. However, RAC1 and RAC1b proteins have a role in tumourigenesis and overexpression in breast cancer and can be activated by TGF‐β.^88^

TGF‐β and the agonist of G‐protein coupled receptor proteinase‐activated receptor‐1 (PAR1) and PAR‐2 are involved in fibrosis and cancer, as well as being involved in the regulation of many processes, such as tumour cell proliferation, migration, invasion and metastasis. TGF‐β provides a regulatory effect on PAR1 expression, and upregulation is associated with enhanced expression of integrin αv and β6 subunits. However, platelet activation, along with the agonist of PAR1, stimulates the release of TGF‐β, which brings EMT in cancer cells. Moreover, TGF‐β and the PAR1 or PAR2 ligand and its receptors interact mutually at different levels of transcription and post‐transcriptional stages. More precisely, they modulate both PAR1 and PAR2 at the transcriptional level. This mutual interaction between PAR1/PAR2 and the TGF‐β signalling leads to feed‐forward loops/brutal cycles of matrix deposition and malignant traits, which aggravate fibrosis and oncogenesis.^89^ Hence, it has been found that the TGF‐β can have crosstalks with various other pathways, such as MAPK, P38 and ERK. This can also be examined further for the simultaneous modulation of these pathways from various natural and synthetic agents.

Higher TGF‐β1 expression was implicated in prostate tumour progression in spite of its growth‐inhibitory effect on normal prostate epithelial and carcinoma cells. It was also reported that TGF‐β1 stimulated the G1‐to‐S transition of the cell cycle in the TSU‐Pr1 prostate cancer cell line. PD98059, a MAPK inhibitor, caused blockade of the MAPK pathway, and also reinstated the growth‐inhibitory role of TGF‐β1 in TSU‐Pr1 cells. Moreover, it was also reported that there was a complete inhibition of negative growth regulation through TGF‐β1 by Smad2, Smad3 or Smad4, revealing a Smad‐independent action. In conclusion, it has been opined that by producing autocrine TGF‐1, prostate carcinoma cells with activated Ras/MAPK pathway may have a selective growth advantage.^90^ The detailed information on the role of TGF‐β and MAPK in the progression of cancer has been described elsewhere.^91^

## 5 TGF‐β INHIBITORS FOR CANCER THERAPY

### 5.1 TGF‐β inhibitors

As described earlier, TGF‐β can act as a tumour promoter or suppressor.^39^, ^82^, ^92^, ^93^, ^94^, ^95^, ^96^ TGF‐β has a prominent role in cancer via the primary phase tumour suppression of neoplasia, and additionally it promotes progression of tumours and metastasis in later stages. The tumour cell growth suppression through TGF‐β is based on the potential of upregulation of cyclin kinase inhibitors. Several malignant tumours can downregulate or mutate the TGF‐βRII receptor or further abnormalities of the TGF‐β signalling pathway.^97^ Several tumours generate large amounts of TGF‐β and are resistant towards its growth inhibition. Simultaneously, TGF‐β generated through tumours can depress antitumour immune responses at cytotoxic T lymphocytes, T‐helper cells, dendritic cells, natural killer (NK), B cells and macrophage levels though enhanced Tr cells.^98^ Downregulation of TGF‐βRIII (betaglycan) is also associated with breast cancer progression.^99^

During late cancer progression phases, TGF‐β acts as a promoter of metastasis through EMT induction. This leads to the enhancement of the invasion of cancerous cells through induction of gene expression, which eases metastatic colonisation of the secondary organ sites, such as bones, lungs, brains and livers.^37^ Despite the fact that information about opposing functions of TGF‐β has existed for a long time within both earlier/later stages of cancer, it is still not clear exactly how and when TGF‐β switches from its role as a tumour suppressor to the metastasis promoter.^78^ In light of the prominent effect of the TGF‐β signalling pathway in tumourigenesis and other diseases, efforts have been made to target TGF‐β signalling in tumours, as well as its microenvironment for the development of chemotherapeutic agents.^64^, ^100^, ^101^, ^102^, ^103^, ^104^

### 5.2 Synthetic TGF‐ β inhibitors

Many TGF‐β signalling antagonists, such as antisense molecules, antireceptor monoclonal antibodies and ligands trap for suppression of the interaction of ligand‐receptor. Inhibitors of TGF‐β receptor kinases aptamers are developed and used in clinical practice.^105^ Many TGF‐β pathway inhibitors are approved and used clinically, and a few small molecules are also emerging within new research (Table 1). Small molecule inhibitors are fundamentally designed to inhibit TGF‐β receptors and are dependent upon imidazole scaffolds or dihydropyrrolopyrazole scaffold. Other inhibitors are based on pyrazole, pyrazolopyridine, triazole, imidazopyridine, isothiazole and pyridopyrimidine scaffolds. Out of the small molecule inhibitors, one drug candidate, galunisertib (LY2157299 monohydrate), established by Eli Lilly, is among the most advanced and promising outcomes in two Phase II trials.^106^ However, the development of this drug was discontinued by Eli Lilly in January 2020 due to financial reasons.

**TABLE 1:** Clinical studies conducted on various synthetic and small molecules as transforming growth factor‐β (TGF‐β) inhibitors studied against different cancers

Drug	Cancer type	Phase	Patient numbers	Outcome	Reference
Fresolimumab (GC1008)	Malignant melanoma and renal cell carcinoma	I	29	Block TGF‐β activation by neutralising TGF‐β1, TGF‐β2 receptors	^106^, ^249^
LY3022859, an anti‐ TGF‐ß receptor Type II (TβRII) monoclonal antibody	Advanced solid tumours	I	14	Inhibit the activation of receptor‐mediated TGF‐β signalling	^250^
Galunisertib	Advanced hepatocellular carcinoma	II	149	Decrease in TGF‐β1 and circulating serum α‐fetoprotein and associated with longer survival	^251^
Galunisertib + gemcitabine	Pancreatic cancer	II	104	Attach to adenosine triphosphate (ATP)‐binding domain of TβR kinases and inhibit receptors’ kinase activity improved overall survival	^252^
AP12009 (trabedersen)	High‐grade glioma	IIb	145	Found superior risk assessment and positive risk‐benefit	^253^
Lanreotide	Pancreatic and intestinal neuroendocrine tumours	III	88	Found beneficial and showed antitumour effects	^254^
Romidepsin	Relapsed peripheral T‐cell lymphoma	II	131	Complete and durable responses with manageable toxicity (approved by Food and Drug Administration)	^255^
Dovitinib	Castration‐resistant prostate cancer	II	44	Modest antitumour activity with controllable toxicities	^256^
M7824	Advanced solid tumours	I	600	Manageable study design and showed efficacy	^257^

An entirely humanised, high‐affinity monoclonal antibody, XOMA089, for the neutralisation of TGF‐β1 and TGF‐β2, has been co‐developed by Novartis and Xoma. Preclinical evidence revealed that XOMA089 exhibited antitumour activity against the growth of squamous cell neck and head carcinoma as well as breast cancer in preclinical models. Additionally, the results also revealed that XOMA089 may have a synergistic effect with programmed cell death protein 1 inhibition.^106^ Another Phase IIb trial was conducted on trabedersen (synthetic TGF‐β2 antisence oligodeoxynucleotide), and temozolomide (or procarbazine/vincristine/lomustine) was conducted on 145 patients with refractory, recurrent glioblastoma multiforme or anaplastic astrocytoma and showed positive results.^107^

A few antibodies, ligand traps and vaccines have also been developed/are under development as TGF‐ β inhibitors with new levels of understanding of the promising therapeutic potential for the development of TGF‐ β inhibitors in cancer therapy. Fresolimumab (GC1008), LY2382770 and P144 are some examples that target the TGF‐β1, β2, β3, TGF‐β1 and TβRI/II complexes, respectively.^108^ However, all these agents are in Phase I or II trials.

## 6 NATURAL COMPOUNDS AS CANCER PREVENTIVE AND THERAPEUTIC AGENTS VIA TARGETING TGF‐β

Within the history of drug discovery, various compounds derived from plants have delivered promising effects in cancer treatment.^109^ Therefore, the inclusion of natural products in cancer treatment in combination therapy (surgery, radiation and chemotherapy) is an active area of interest.^110^ In a recent comprehensive analysis by Newmann and Cragg,^9^ the authors clearly indicated natural products as the best options to search for novel agents/active templates that offer tremendous potential for drug discovery for a range of human diseases, including cancer. Several studies have reported that a diverse range of dietary and non‐dietary compounds possess an important role in cancer prevention and therapy.^111^

### 6.1 Methodology for literature search and selection

**FIGURE 2:**
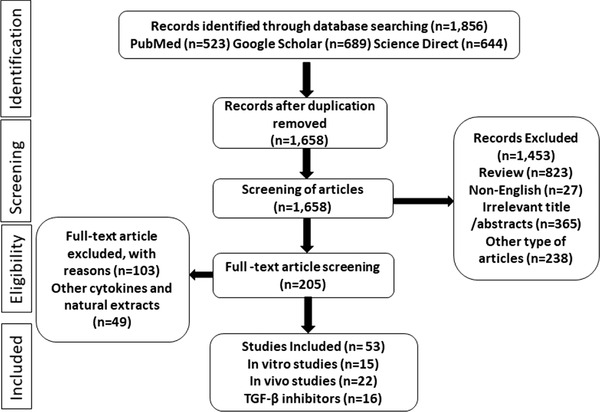
Preferred Reporting Items for Systematic Reviews and Meta‐Analysis diagram showing literature search and selection strategy

Various authentic and scholarly databases, such as PubMed, Science Direct, Google Scholar and Scopus, were explored to search and obtain primary literature for this study. The Preferred Reporting Items for Systematic Reviews and Meta‐Analysis criteria^112^ was followed for the review (Figure 2). Unpublished results, conference abstracts, books and articles written in other languages than English were excluded. For the literature search, major keywords, such as phytochemicals, natural products, extracts, TGF‐β, cancer, apoptosis, proliferation, signalling pathway, treatment, prevention, in vitro, in vivo and clinical studies, were used in various combinations. In addition, bibliographies of primary literature were studied to gather additional appropriate articles. We have also used the Boolean information retrieval method^113^, ^114^ using TGF with the ‘AND’ operator. The last search was conducted in August 2021.

### 6.2 TGF‐β signalling pathway modulation by anticancer phytochemicals

Researchers have performed various experiments to understand the mode of action of several natural products on the TGF‐β signalling pathway in various models of cancer through in vitro and in vivo studies. The following sections summarise the main findings regarding different natural products, which were evaluated for their potential anticancer effects through interference with the TGF‐β pathway.

#### 6.2.1 Arjunolic acid

**FIGURE 3:**
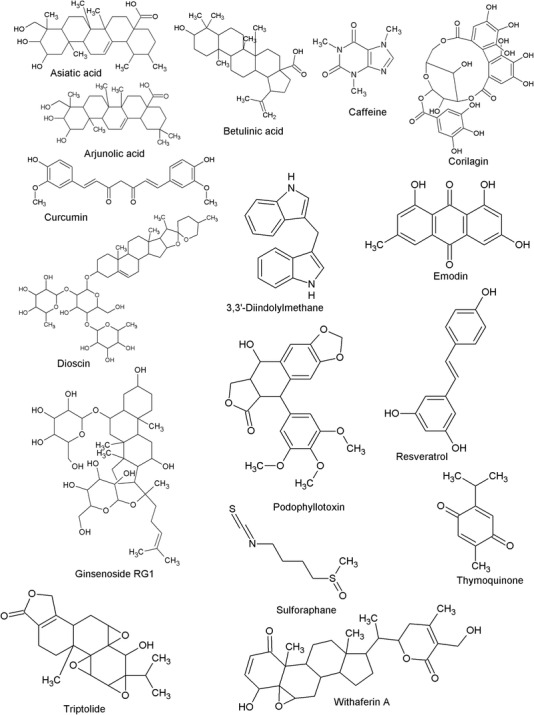
Chemical structures of bioactive phytochemicals affecting TGF‐β signalling in cancer

Arjunolic acid (Figure 3) is a triterpenoid saponin, isolated initially from *Terminalia arjuna* as well as from other plant species, such as *Combretum nelsonii* and *Leandra chaeton*.^115^ A study was performed to examine the antitumour effect of 100 and 250 mg/kg of arjunolic acid in mice bearing Ehrlich ascites carcinoma, and it was found that arjunolic acid was able to reduce the volume of tumours as well as decrease their cell count and viability. It was also reported that arjunolic acid increased cellular toxicity. Additionally, arjunolic acid reduced interleukin (IL)‐1β, TNF‐α, TGF‐β and TGF‐β Type I receptors and levels of latency‐linked peptide related with elevated IL‐10. Therefore, arjunolic acid was found to possess antitumour activity against Ehrlich ascites carcinoma cells via enhancement of cytotoxicity and apoptosis. This effect was mediated by the partial blocking of TGF‐βR1 and also by its impact upon the levels of inflammatory cytokine^116^ (Table 2). Although the study showed an inhibition of TGF‐β by arjunolic acid, it has not been explored how arjunolic acid caused this effect.

**TABLE 2:** Natural products conferring anticancer effects via regulation of TGF‐β signalling in vitro

**Compound/ extract**	**Concentration tested**	**Cell line used (cancer type)**	**Anticancer effects**	**Effect on TGF‐β signalling**	**Reference**
Arjunolic acid	20–120 μM	Ehrlich ascites carcinoma (breast cancer)	↑Cytotoxicity, ↑apoptosis	Blocked TGF‐β1R1	^116^
Asiatic acid	5–80 μM	A549 (lung cancer)	↓Cell viability	Inhibited TGF‐β1‐induced cell invasion and migration	^119^
Betulinic acid	1–15 μM	RKO and SW480 (colon cancer)	↓Cell growth, ↑ apoptosis	Downregulated Sp1, Sp3 and Sp4	^122^
Caffeine	.2–.8 mM	MDA‐MB‐231 (breast cancer)	Stabilise active breast stromal fibroblasts and ↓metastatic potential, ↓SDF‐1, and ↓ matrix metalloproteinase 2 (MMP‐2) expression	Suppressed TGF‐β expression	^134^
Corilagin	20–80 μM	SKOv3ip, Hey and HO‐8910PM (ovarian cancer)	Arrested G2/M phase, ↑apoptosis, ↓cytokine, ↓cyclin B1, ↓Myt1, ↓phospho‐cdc 2	Inhibited secretion of TGF‐β and blocked the TGF‐β‐induced Snail stabilisation	^139^
Curcumin	10 μM	A549 (lung cancer)	↓Metastasis via miRNA gene network, and ↓ mitogen‐activated protein kinase (MAPK), ↓Wnt signalling pathways.	Downregulated of TGF‐β	^145^
Curcumin	12.5–50 μM	BCPAP cell line (thyroid cancers)	↑E‐cadherin and ↓vimentin expression and ↓cell attachment, ↓migration, ↓progression, ↓ small mothers against decapentaplegic homolog 2 (Smad2) and Smad3 phosphorylation	Inhibited TGF‐β1	^147^
Curcumin	10–30 μM/ml	PANC‐1 cell line (pancreatic cancer)	↓Cell proliferation, ↓cell migration, ↑apoptosis	Inhibited TGF‐β1 signalling pathway, reversed epithelial‐mesenchymal transition (EMT) of TGF‐β1 via Hedgehog signalling pathway	^148^
Curcumin + endoxifen + betaestradiol	8.5–17 μM	MCF‐7 (breast cancer)	↓Cell viability, ↑vimentin, and ↑E‐cadherin expression.	Endoxifen increased mRNA expressions of TGF‐β1; however, curcumin decreased TGF‐β1 mRNA expressions	^238^
Curcumin + emodin	15–25 μM	SiHa and HeLa (cervical cancer)	↓Cell migration, ↓EMT markers	Downregulated TGF‐β, ↓TGF‐β receptor II expression; ↓Wnt/β‐catenin signalling in the presence of TGF‐β↓ P‐Smad3, Smad4, ↓cyclin D1, p21 and Pin1	^146^
3,3′‐Diindolymethane	10–40 μM	MCF‐7 and HCC38 (breast cancer)	↓Cell migration, ↓Smad2/3, ↓ extracellular signal‐related kinase 1/2	Suppressed TGF‐β/ tumour necrosis factor‐α signalling pathway	^159^
Dioscin	.5–4 μM	A549 (lung cancer)	↓Cell growth, and ↓cell proliferation	Suppressed the EMT induced by TGF‐β, ↑ E‐cadherin and N‐cadherins expression induced by TGF‐β	^171^
Dioscin (disogenin)	.5–2 μM	HepG2 cells (hepatocellular carcinoma)	↓Cell division, invasion and migration and ↓MAPK pathway, ↓cell proliferation	Reversed growth‐promoting activity of TGF‐β	^170^
Emodin	15–25 μM	SiHa and HeLa (cervical cancer)	↓Cell migration, and ↓ EMT markers	Downregulated TGF‐β signalling pathway, ↓TGF‐β receptor II expression; ↓Wnt/β‐catenin signalling pathway in the presence of TGF‐β↓ P‐Smad3, Smad4, ↓cyclin D1, p21 and Pin1	^146^
Ginsenoside Rb2	20–40 μM	Ishikawa cell lines and HEC1A (endometrial cancer)	↓Growth of cells and metastasis, ↑E‐cadherin levels, ↓vimentin	Decreased TGF‐β and Snail levels	^180^
Ginsenoside Rb2	.1 mg/ml	PC3 (prostate cancer)	↓Cell proliferation and ↓invasion by regulation of cell‐cycle controllers and MMPs	Activated TGFβ receptor signalling	^181^
Ginsinoside Rb2	.1–10 μg/ml	HCT116 and SW620 (colorectal cancer)	↓EMT, adhesion, growth, metastasis of colorectal cancer cell (CRC)	Inhibited TGF‐β1 expression. Docking simulation showed binding of ginsinoside Rb2 to TGF‐β1 and disrupted TGF‐β1 dimerisation	^179^
Ginsinoside Rb2	25–100 μg/ml	HNE1 and CNE2 nasopharyngeal carcinoma (NPC)	↓Invasion and migration ability of NPC cells and the EMT process	Reversed TGF‐β induced morphological conversion and alteration in marker proteins and EMT	^182^
Podophyllotoxin	1.56 μM	Hepatocellular carcinoma cell lines (liver cancer)	↓Migration and invasion and ↓ MMP.	No effect due to TGF‐β1 but due to p53/ phosphatidylinositol 3‐kinases (PI3K)/ protein kinase B (Akt)/ mammalian target of rapamycin pathway	^186^
Resveratrol	6.25–200 μM	LoVo (colorectal cancer)	↓EMT	Decreased TGF‐β1; effect produced via TGF‐β1/Smads signalling pathway‐mediated expression of Snail/E‐cadherin	^194^
Resveratrol	5–80 μmol/L	PLA‐802 (rhabdomyo sarcoma)	↓Cell growth, ↓Smad4 expression at protein and mRNA levels, ↓cells in the S phase, arrested G0/G1 transition	Decreased TGF‐β1	^195^
Resveratrol	50 μM	Tamoxifen‐resistant MCF‐7 (breast cancer)	Reversed EMT	Suppressed production of endogenous TGF‐β; acts as a chemosensitiser by TGF‐β/Smad signalling, ↓ Smad cascade	^196^
Resveratrol	25 μmol/L	A549, NCI H23 and NCI H460 (lung cancer)	↓Proliferation by cell cycle arrest, ↑apoptosis, ↓Smad activators, ↓Smad2 and Smad4, ↓mRNA level, ↑Smad7	Blocked the nuclear signalling of TGF‐ β pathway and altered the intracellular Smad signalling of the TGF‐β	^197^
Sulforaphane	10–80 μM	HepG2 (hepatocellular carcinoma)	↓Cell invasion, ↓cell migration, ↓cell proliferation	Inhibited TGF‐β induced EMT via ROS‐dependent pathway	^207^
Thymoquinone	5–20 μM	MCF 7 and MDA‐MB‐231 (breast cancer)	↓Cell proliferation, ↓Bcl2, ↑p53	Restored the basal level of TGF‐ β	^213^
Triptolide	25–100 nM	HCT116, CRC, HT29 and SW620 (colon cancer)	↓Cell migration, ↓cell proliferation	Suppressed TGF‐βRI ans II	^219^
Withaferin A	.8–1.2 μM	Caski and SK‐Hep1 (metastatic cancer)	↓Invasive property, ↓migratory ability, ↓MMP‐9 expression via pAkt signalling pathway suppression	Inhibited TGF‐β induced phosphorylation of Akt	^225^
Withaferin A	2 μM	MDA‐MB‐231, MCF‐10A and MCF‐7, cells (breast cancer)	Reversed biochemical features of EMT	Inhibited TGF‐β‐induced EMT and migration	^224^
*Withania somnifera* root extract	500 nM	MDA‐MB‐231, HCC1806, T47D, MCF‐7, Hs578‐T and MDA‐MB‐468 (breast cancer)	Potential association was found between vimentin expression and cytotoxicity	TGF‐β inhibits vimentin expression at the protein level but not at mRNA level	^228^
*Andrographis paniculata* herb extract	800 μg/ml	EC‐109 (oesophagal cancer)	↑Apoptosis, ↓proliferation, ↓metastatic, ↓drug resistance and ↓intercellular adhesion	TGF‐β gave negative feedback	^258^
*Momordica charantia* leaf extract	25–50 mg/ml	PLS10 cell line (prostate cancer)	↓Progression by anti‐invasive effects	Induction of TGF‐β has not reported	^259^

#### 6.2.2 Asiatic acid

Asiatic acid (Figure 3), an ursane‐type pentacyclic triterpenoid molecule, is mainly obtained from *Centella asiatica*.^117^ In a study conducted with asiatic acid in combination with naringenin, a potential inhibition of translation and phosphorylation of Smad3 was shown; however, a restoration of Smad7 expression was also reported.^118^ The effect was mediated by the promotion of NK cell differentiation, maturation and cytotoxicity via Id2/IRF2‐associated mechanisms. In addition, naringenin and asiatic acid exhibited an additive‐type effect on TGF‐β1/Smad3 signalling inactivation and reduced lung carcinoma as well as melanoma growth.^118^ In another study, asiatic acid increased protein and mRNA expression levels of E‐cadherin, and reduced expression levels of N‐cadherin, snail family transcriptional repressor (Snail) and β‐catenin in A549 cells treated with TGF‐β1, hence inhibiting TGF‐β1‐induced EMT in lung cancer.^119^

#### 6.2.3 Betulinic acid

Betulinic acid (Figure 3) is a triterpenoid phytoconstituent reported for the growth inhibition of different tumours.^120^, ^121^ Betulinic acid was evaluated against prostate cancer using SW480 and RKO colon cancer cells and also by utilising the athymic nude mice xenograft model (Table 3). The study exhibited that betulinic acid has the ability to inhibit tumour growth and induce apoptosis by decreasing the expression of various transcription factors, namely, Sp1, Sp3 and Sp4, as well as decreasing levels of the survivin, p65 subunit of NF‐κB and cyclin D1.^122^ It was also reported that betulinic acid did not have the ability to inhibit the production of TGF‐ β but butein did.^117^ However, it is notable a substantial amount of studies are not available for understanding the effect of betulinic acid against different cancer models.

**TABLE 3:** In vivo anticancer activities of natural products affecting TGF‐β signalling

**Compound/extract**	**Cancer type**	**Animal model**	**Dose (mg/kg)**	**Anticancer effect**	**Effect on TGF‐β signalling**	**Reference**
Betulinic acid	Colon cancer	RKO xenograft athymic mice model	25	↓Tumour growth, ↓tumour volume	Reduced expression of Sp1, Sp3 and Sp4	^122^
Ginsenoside Rh2	Prostate cancer	PC3‐luc glioblastoma imaging by bioluminescence	1	↓Cell growth, ↓cell proliferation and invasion of prostatic cancer	Activated TGFβ signalling	^181^
Ginsenoside Rb2	Colorectal cancer	HCT116 xenograft tumour model	10	↓Tumour volume, ↓tumour growth	Inhibition of EMT and inhibited TGF‐β1, TGFβRI and TGFβRII	^179^
Polyphyllin	Gastric cancer	GC7901/DDP xenograft studies	1	↓Tumour growth, ↓tumour weight	Antagonised the facilitative effects of TGF‐β1	^226^
Resveratrol	Colorectal cancer	LoVo cells in vivo imaging in mice by tail vein injection	150	Reduced metastatic lesions	Inhibited TGF‐β1 effects on EMT	^194^
Resveratrol	Lung cancer	MDA231 xenograft‐bearing mouse model	40	↓Lung metastasis of breast cancer, ↓tumour growth	Inhibited EMT induced by TGF‐β1‐via PI3K/Akt, Smad and MMP regulation	^198^
Sulforaphane	Liver cancer	HepG2 xenograft mouse tumour model	50	↓Tumour growth, ↓tumour volume ↓Proliferation, migration and invasion, ↓EMT via reactive oxygen species‐dependent pathway	Suppressed TGF‐β‐induced EMT	^207^
Thymoquinone	Hepatocellular carcinoma	Thioacetamide induced liver cancer	20	↓Growth and progression through ↓ oxidative stress, induction of TNF‐related apoptosis‐inducing ligand (TRAIL)‐mediated apoptosis	Decreased hepatic TGF‐β1 mRNA level by 1.8‐fold	^214^
Thymoquinone	Renal cell carcinoma	Tumour xenograft model male C57BL/6 on mice	10 and 20	Prevents 786‐O‐SI3 cells transfer to lungs in mice,↓invasion, ↓cell movement and production, cell adhesion and cytoskeletal reorganisation	Suppressed TGF‐β1	^215^
Triptolide	Melanoma	Melanoma bearing mice model	.15	↓Tumour growth by reduction in proportion of regulatory T cells and Foxp3 level in lymph node and spleen in tumour‐bearing mice	Decreased TGF‐β, IL10 and vascular endothelial growth factor	^221^
Withaferin A	Breast cancer	MMTV‐neu transgenic mouse model using female athymic mice	4	↓Growth of cancer cells and migration with ↓vimentin protein expression	Reversed the EMT induced by TGF‐β	^224^
*Withania somnifera* root extract	Breast cancer	Human xenograft and mouse mammary carcinoma models	4, 8	↓Motility, ↓invasion of cancer cells and disturbed vimentin morphology and ↓tumour volume	Inhibited EMT induced by TGF‐β	^228^

TGF‐receptors are translocated from lipid raft/caveolae microdomains to non‐caveolae microdomains by betulinic acid, with no change in total TGF‐receptor levels. TGF‐receptor translocation mediated by betulinic acid is rapid, and it correlates with TGF‐driven plasminogen activator inhibitor‐1 (PAI‐1) reporter gene activation and growth inhibition in Mv1Lu cells. The betulinic acid‐induced TGF‐β receptor translocation is also rapid and correlates with the growth inhibition and TGF‐β‐induced PAI‐1 reporter gene activation in Mv1Lu cells.^54^

Another study on a bioactive subfraction from *Mesua ferrea* stem bark was performed on HCT 116 colon cancer cells. The betulinic acid fraction was the major active component examined. This study has reported that multiple pathways were involved in the anticancer activity of the standardised fraction. The mechanism behind the effect was attributed to the downregulation of Wnt, epidermal growth factor receptor and HIF‐1α along with a simultaneous upregulation of TGF‐β, p53 and Myc/Max signalling pathways.^123^ This shows that betulin shows its anticancer effect by simultaneous modulation of multiple pathways, including TGF‐β signalling.

#### 6.2.4 Caffeine

Caffeine (Figure 3) is naturally found in over 60 different plants as well as various beverages and food items, including tea, chocolate, cocoa beans, kola nuts and guarana berries.^124^ Caffeine was found potentially effective in the treatment of many cancers, such as colon, bladder and pancreatic cancers.^125^, ^126^, ^127^, ^128^ It is easily available as an over‐the‐counter drug for pain relief and also possesses anti‐inflammatory and analgesic effects.^129^, ^130^ Caffeine suppresses oxidative stress and inflammation, improves insulin sensitivity, inhibits angiogenesis and has a protective role in liver cancer.^131^ Various studies convey that consumption of caffeine reduces the risks of different types of cancer as it affects cell cycle progression, influences apoptosis and inhibits angiogenesis and migration by antagonising adenosine receptors.^132^

In recent years, caffeine has emerged as a potentially active compound for its preventive and therapeutic role in cancer. Mechanistically, caffeine affects the progression of the cell cycle at checkpoints, persuades apoptosis and hinders drug efflux from cells.^131^, ^132^, ^133^ Caffeine showed potent anticancer effects through TGF‐β, as it upregulated various tumour suppressor proteins, such as p16, p21, p53 and caveolin‐1in breast stromal fibroblasts. It also inhibited the metastasis of cancer‐associated fibroblast cells (CAF) through phosphatase and tensin homolog‐dependent ERK1/2 and by Akt activation. It inhibited the release of vascular endothelial growth factor (VEGF) A in CAF cells and inhibited the pro‐angiogenic activity of breast cancer cells.^134^

Caffeine inhibits TGF‐β‐induced CTGF production in rat hepatocytes via stimulation of TGF‐β effector Smad2 degradation, inhibiting phosphorylation of Smad3, and PPARγ‐receptor upregulation.^135^ Caffeine is able to upregulate various tumour suppressor proteins, such as p21, p16, p53 and Cav‐1, and is also able to reduce expressions of IL‐6, SDF‐1, TGF‐β and matrix metalloproteinase 2 (MMP‐2), as well as downregulate α‐SMA. The study has concluded that caffeine may suppress the pro‐carcinogenic actions of active stromal fibroblasts, making it a safe and efficient way to prevent breast tumour growth and recurrence.^134^ A similar purine alkaloid to caffeine, theacrine, was reported to attenuate EMT, cell adhesion, invasion and migration induced by TGF‐β in MDA‐MB‐231 cells. The results indicated that theacrine inhibited breast cancer cell metastasis through EMT process reversal.^136^

In a clinical study, after 5 months of green tea and chemotherapy administration, the TGF‐β and IL‐10 serum levels decreased in patients and control groups during the green tea intake period. Green tea appears to be able to modify circulating Tregs in chronic lymphocytic leukaemia patients in the early stages of the disease. This can help to keep lymphocytosis under control while also preventing disease development.^137^

#### 6.2.5 Corilagin

Corilagin (β‐1‐O‐galloyl‐3,6‐(R)‐hexahydroxydiphenoyl‐d‐glucose, Figure 3) is an ellagitannin compound isolated from *Phyllanthus niruri* L. as well as other plants, such as *Caesalpinia coriaria*. Corilagin has been recently examined for several therapeutic activities, including cancer.^138^ Corilagin was studied against ovarian cancer cell lines, namely, SKOv3ip, HO‐8910PM and Hey, and also in a xenograft tumour model.^139^ The study demonstrated that corilagin has a specific targeting effect on TGF‐β secretion, and thus was able to block non‐canonical ERK/Akt and canonical Smad pathway activation.

When evaluated in ovarian cancer cell lines (OC316, OVCAR5 and HOPMSnail), corilagin treatment was able to downregulate Snail expression. TGFβ‐enhanced Snail was also blocked in HOPM‐Snail cells. Thus, it was revealed that corilagin not only showed its effect by acting via distinct pathways, such as Snail inhibition but also as an apoptosis inducer, such as chemotherapeutic drugs.^140^ In another similar study, corilagin inhibited TGF‐β secretion into the culture supernatant of different ovarian cancer cell lines and inhibited TGF‐β‐induced Snail stabilisation. TGF‐β production was not reduced in cancer cells treated with paclitaxel, demonstrating that corilagin specifically targeted TGF secretion.^139^

#### 6.2.6 Curcumin

Curcumin ((1E,6E)‐1,7‐bis(4‐hydroxy‐3‐methoxyphenyl)‐1,6‐heptadiene‐3,5‐dione Figure 3) is extracted from the rhizome of *Curcuma longa* of family Zingiberaceae. Curcumin has aromatic parts, such as an O‐methoxy phenolic and β‐dicarbonyl group. There are various biological properties and therapeutic benefits of curcumin that can be utilised for the treatment of chronic human disease, as it possesses antioxidant, anti‐inflammatory, anticancer, antidiabetic, immune‐regulatory, cardiovascular protective, neuroprotective and hepatoprotective effects.^141^, ^142^, ^143^

Curcumin possesses anticancer effects when studied in pre‐clinical and clinical conditions.^16^, ^144^ Curcumin delivers its anticancer properties by stimulation of apoptotic pathway in cancer cells along with suppression of tumour microenvironment, such as inflammation, angiogenesis and tumour metastasis.^16^ Several studies were performed to evaluate the potential of curcumin to treat various cancers, such as thyroid, lung, cervical, pancreatic and breast cancers, through modulation of the TGF‐β pathway. In lung cancer, curcumin was able to inhibit metastasis and suppress cancer cell proliferation via interference with many other pathways along with TGF‐β, such as MAPK or the Wnt signalling pathway.^145^ The TGF‐β signalling pathway was downregulated by curcumin via a decrease in the expression of TGF‐β receptor II, P‐Smad3 and Smad4 and reduced TGF‐β‐induced invasion and migration in the HeLa and SiHa cells lines. Additionally, it inhibited p21, cyclin D1 and Pin1, with Slug and Snail in human cervical cancer cell lines.^146^ The study^146^ showed that curcumin and emodin exhibited a synergistic effect and inhibited cell migration and TGF‐β activated the Wnt/β‐catenin signalling pathway.

Curcumin increased E‐adherin expression while repressing vimentin expression and also suppressed cell attachment, migration and progression in BCPAP thyroid cancer cells. It was also found to inhibit TGF‐β1‐mediated Smad2 and Smad3 phosphorylation and also inhibited TGF‐β1‐induced EMT via Smad2/3 downregulation.^147^ Sun et al.^148^ studied the effect of curcumin against pancreatic cancer and showcased that curcumin repressed cell proliferation, decreased cell migration, invasion, induced apoptosis and reversed EMT of TGF‐β1‐stimulated PANC‐1 cells via Hedgehog signalling pathway inhibition.^148^

In a notable study, curcumin in combination with doxorubicin reduced the survival and proliferation of neuroblastoma cells by inducing apoptosis via p53 and p21 upregulation.^149^ Another study emphasised the importance of time and demonstrated that a combination of curcumin and doxorubicin promotes apoptosis via Bcl‐2 downregulation, Bax and acaspase‐9 overexpression in a concentration‐ and time‐dependent way.^150^ In another study, curcumin and emodin synergistically suppressed cell migration and population in HeLa and SiHa cells. TGF‐ β also stimulated the Wnt/β‐catenin signalling pathway in HeLa cells, and emodin and curcumin decreased β‐catenin, hence suppressing the pathway. Therefore, curcumin and emodin can be used synergistically for the treatment of cervical cancer.^146^

HCT116/oxaliplatin (OXA), an oxaliplatin‐resistant cell line, was effectively developed, and OXA combined with curcumin lowered OXA resistance in vitro. Furthermore, the combination treatment reduced p‐p65 and Bcl‐2 expression while increasing activated caspase‐3 levels. Curcumin also prevented EMT by regulating the TGF/Smad2/3 signalling pathway. Curcumin could also reduce OXA resistance in colorectal cancer cells in an in vivo investigation.^151^ Curcumin blocked the TGF‐β and PI3K/Akt signalling pathways, which are both involved in doxorubicin‐induced EMT. Curcumin also improved doxorubicin's antiproliferative actions in triple‐negative breast cancer cells and showed that doxorubicin in conjunction with curcumin could be a viable treatment option for triple‐negative breast cancer.^152^

A quercetin and curcumin combination was evaluated against K562/CCL‐243 chronic myeloid cell lines and found to be effective on various genes attributed to the p53, NF‐κβ and TGF‐α pathways. For instance, the combination exhibited a downregulatory effect on IFN‐ γ AKT1, CDKN1B and an upregulatory effect on CDKN1A, BTG2 and FAS. A multitargeted therapy for chronic myeloid leukaemia cells without impacting healthy cells may be provided by the downregulation of CDKN1B, AKT1, IFN‐ α and upregulation of gene and protein expressions of BTG2, CDKN1A and FAS.^153^ The combination of cisplatin and nanocurcumin reduced the volume and weight of ovarian tumours significantly. There was also a decrease in Ki67, TGF‐β, PI3K and Akt phosphorylation expression. JAK expression, STAT3 phosphorylation and IL‐6 concentrations were all lowered when cisplatin and nanocurcumin were combined. Overall, nanocurcumin, when given as a co‐treatment with cisplatin, inhibited proliferation in ovarian cancer models by downregulating the PI3K/Akt and JAK/STAT3 signalling pathways.^154^ However, one in vivo study showed no involvement of TGF‐β when curcumin was investigated against tumourigenicity assay using an athymic nude mouse model. This suggests that more confirmatory studies are required to understand the effect of curcumin on TGF‐β.^155^

#### 6.2.7 3,3′‐Diindolymethane (DIM)

DIM (Figure 3) is a dimeric condensation product of indole‐3‐carbinol and a potent anticancer compound obtained from several vegetables of the genus *Brassica*, including broccoli, cabbage and Brussels sprouts. It is considered to exhibit anticancer properties by affecting cell proliferation, autophagy, endoplasmic reticulum stress, the cell cycle and apoptosis through different signalling mechanisms.^156^ DIM selectively kills cancer cells without toxic exposure to normal cells.^157^ DIM was examined against human endometrial cancer cells and exhibited a cytostatic effect. The mechanistic investigation revealed that this effect was associated with the enhancement of TGF‐α.^158^ Another study has shown that DIM has the potential to inhibit breast cancer migration through inhibition of the TGF‐β and TGF‐α signalling pathways, which was associated with the suppression of EMT.^159^ In a recent study, DIM was evaluated against MCF‐7 breast cancer cells, and it was reported that DIM significantly reduced the TNF‐α/TGF‐β‐induced breast cancer cells migration. Furthermore, the results demonstrated that DIM efficiently suppressed EMT processes by the inhibition of TNF‐α/TGF‐β‐associated signalling pathways in breast cancer cells. Hence, it can be stated that DIM has the potential to serve as a candidate for breast cancer therapy.^159^

#### 6.2.8 Dioscin

Diosgenin (Figure 3) is a steroid saponin extracted from the tuber of various species of *Dioscorea*. It is frequently used in the commercial synthesis of estrogen, progesterone, cortisone and steroids. Diosgenin displays many beneficial properties, such as antioxidant,^160^ anti‐inflammatory,^161^ hypoglycemic,^161^ hypolipidemic^162^ and antidiabetic^163^ effects. Additionally, it is effective against a variety of cancers and has shown a pro‐apoptotic effect.^164^ It has been demonstrated that diosgenin induces anticancer activity mainly by its antiproliferative property, induces apoptotic cell death, and avoids the formation of malignant tissue by arresting the cell cycle phases.^164^, ^165^, ^166^, ^167^ Dioscin, a natural derivative of diosgenin, is used for the treatment of breast cancer.^168^, ^169^

Dioscin exhibited inhibition of proliferating HepG2 hepatocellular cancer cells. Low concentrations of dioscin reversed the growth‐promoting effect on HepG2 cells induced by TGF‐β1. Furthermore, it suppressed TGF‐β1‐induced proliferation of HepG2 cells. The compound also inhibited the migrations and invasive property of cells, and treatment with dioscin increased the expression of various proteins, such as claudin‐1 and E‐cadherin. It decreased the expression of N‐cadherin, vimentin and slug. Dioscin inhibited the MAPK pathway, which has a significant role in the growth of cancer cells and metastasis, as well as inhibiting the EMT malignant transformation triggered by TGF‐β1 in HepG2 liver cancer cells.^170^ When evaluated against lung cancer cell lines, dioscin inhibited cell growth and suppressed cell proliferation in a concentration‐dependent manner. The compound was also found to suppress the EMT induced by TGF‐β in A549 lung cancer cells. Additionally, it increased the expression of E‐cadherin and N‐cadherin, and decreased cell migration and invasion of TGF‐β1‐induced A549 lung cancer cells.^171^

#### 6.2.9 Emodin

Emodin (1,3,8‐trihydroxy‐6‐methylanthraquinone, Figure 3) is a natural anthraquinone derivative, mostly present in several Chinese medicinal herbs. It is isolated from many plant parts, such as roots and barks of plants belonging to the Rubiaceae, Rhamnaceae, Fabaceae and Polygonaceae families. It exerts various pharmacological effects, including antibacterial, antiviral, antiulcerogenic, diuretic vasorelaxant and antitumourigenic properties.^172^ Emodin was studied against human cervical cancer cell lines, and it was found that emodin was able to downregulate the TGF‐β signalling pathway.^146^

Emodin in combination with 3′‐azido‐3′‐deoxythymidine was found to exhibit a synergistic effect on cell apoptosis and proliferation and downregulated NF‐κB, TGF‐β mRNA, and Bcl‐2 proteins in the concentrated leukaemia stem cells (KG‐la cells). The synergic effect was mechanistically associated with Bcl‐2 activation inhibition and TGF‐β and NF‐κB downregulation.^173^

#### 6.2.10 Ginsenosides

Ginsenosides (Figure 3) are triterpene saponins obtained from Panax ginseng, commonly known as Chinese ginseng of the family *Araliaceae*. Ginsenosides were first isolated from this family in 1963^174^ and have shown substantial potential in the treatment of cancer. Furthermore, ginsenosides are well‐known for their cardioprotection, antiobesity, antidiabetic, immunomodulation, neuroprotection, antimicrobial action, sexual potentiating and antitumour activities.^175^ They have many beneficial effects, which explains their use as a complementary or an alternative form for the treatment of various cancers, such as breast, colorectal, endometrial and nasopharyngeal carcinoma.^175^, ^176^, ^177^, ^178^ They exhibit anticancer effects by inhibiting the proliferation of cancer cells through oxidation prevention and by inducing apoptosis and autophagy.^177^

To evaluate the anticancer effect in colorectal cancer through modulation of TGF‐β, a docking study was performed between the ginsenoside Rb2 and TGF‐β complex. The docking score was −9.667 by binding to the hydrophobic pocket of TGF‐β1 and partially overlapping with the TGF‐binding site and finally disrupting the TGF‐β1 dimerisation. Further, western blot analysis also showed inhibition of the expression of TGF‐β1 in HCT116 and SW620 cells. Ginsenoside Rb2 was able to inhibit EMT and upregulate the expression of E‐cadherin as well as downregulate the function of N‐cadherin and vimentin.^179^

Ginsenoside Rb2 was evaluated on HEC1A and Ishikawa cell lines for endometrial cancer. It displayed growth inhibitory effects on the cancer cells by reversing EMT‐induced changes in cell mobility and metastasis, and through further western blot analysis, it displayed an improvement in the E‐cadherin levels while the expression level of vimentin along with TGF‐β and Snail declined. The result suggested that ginsenoside can deliver the anticancer effect by inducing apoptosis and by inhibiting EMT.^180^ Another study was conducted to examine the effect of ginsenoside Rb2 on PC3 prostate cancer cell lines. The results revealed that the compound activated TGF‐β receptor signalling and inhibited cell proliferation and invasion by regulation of cell cycle controllers and MMPs.^181^ A recent study showed the potential suppressive effect of ginsenoside Rg3 against nasopharyngeal carcinoma (NPC) cells, where Rg3 inhibited migration and invasion ability of NPC cells and the EMT process. Additionally, Rg2 altered marker proteins of EMT and affected TGF‐β‐induced morphological transition.^182^

#### 6.2.11 Podophyllotoxin

Podophyllotoxin (Figure 3), a non‐alkaloidal antimitotic lignan, is extracted from the roots and rhizomes of podophyllum species. The compound also has different derivatives, such as teniposide and etoposide, which have obtained scientific focus due to their widespread biological properties, including anthelminthic, antiviral and antineoplastic effects.^183^ The podophyllotoxin, along with its derivatives, is widely used for the effective treatment of breast, lung, ovary and stomach cancer.^184^, ^185^ Upon evaluation of liver cancer cells (Bel‐7402, HepG2 and HCCLM3), podophyllotoxin was found to reverse the effect of EMT without having any effect from TGF‐β1. In addition, a significant inhibition in metastasis and invasion on liver carcinoma cells was also recorded.^186^

The polyamidoamine dendrimer‐conjugated podophyllotoxin treatment substantially decreased NF‐κB and IL‐6 in mice tissue and serum, respectively. A significant reduction was reported in liver fibrous tissue deposition, which was confirmed by declined levels of mRNA and TGF‐β expressions in the liver. Thus, DPODO treatment suppressed hepatocellular carcinoma progression via modulation of fibrogenic and inflammatory factors.^187^

#### 6.2.12 Resveratrol

Resveratrol (3,4,5‐*trans*‐trihydroxy‐stilbene, Figure 3) is a naturally occurring phytoalexin, belonging to the stilbene family. It is isolated from white hellebore *Polygonum cuspidatum* roots as well as many other plant families, such as Fabaceae, Gnetaceae and Cyperaceae. Various food products, including grapes, wine, mulberry and peanuts, contain this compound.^188^ Resveratrol has the capability to target cancer cells at their initiation, promotion and progressing stages. It displays chemopreventive and chemotherapeutic effects by regulating the signal transduction pathways that manage cell division, initiates apoptosis, reduces inflammation, angiogenesis and inhibition of metastasis and invasion of human tumour cells.^15^, ^189^

Resveratrol has many medicinal properties, including antioxidant, anticancer, cardioprotective, immunomodulatory, antihypertensive, anti‐inflammatory, antimicrobial, antidiabetic and neuroprotective activities; hence, it can protect against many diverse chronic diseases.^15^, ^190^, ^191^, ^192^, ^193^ Various in vitro and in vivo studies have established the potential effect of resveratrol against colorectal cancer, and it was found effective in inhibition of EMT by TGF‐β1 and suppressed invasive and metastatic properties. Additionally, resveratrol was able to enhance the expression of E‐cadherin and suppress the expression of vimentin. It also suppressed the invasive and migratory properties and inhibited the TGF‐β1/Smad pathway when examined in the LoVo cell line.^194^

Resveratrol was evaluated against rhabdomyosarcoma soft tissue malignant tumour cells and was found to decrease cell growth, reduce cells in the S phase, arrest G0/G1 transition and reduce TGF‐β1and Smad4 expression at the protein and mRNA levels in PLA‐802 human alveolar rhabdomyosarcoma cell.^195^ Shi et al.^196^ studied the effect of resveratrol on MCF‐7 breast cancer cells and found that resveratrol reversed EMT and has the ability to overcome tamoxifen resistance. The effect of resveratrol was meditated by TGF‐β/Smad signalling, and resveratrol was found to suppress the production of endogenous TGF‐β and downstream Smad cascade. Whyte et al.^197^ studied the effect of resveratrol against A549, NCI H23 and NCI H460 lung cancer cell lines and found that resveratrol inhibited A549 cell proliferation via cell cycle arrest, apoptosis induction and alteration of the intracellular Smad signalling of the TGF‐β pathway. Following resveratrol treatment, Smad activators, namely, Smad2 and Smad4, were downregulated at the mRNA level, Smad7 was upregulated and Smad2/Smad3 at the protein levels blocked nuclear signalling of the TGF‐β pathway.

Resveratrol was reported to inhibit the migration of MDA231 cells via TGF‐β1‐induced EMT reversal and also inhibited the lung metastasis in the MDA‐MB‐231 xenograft mouse tumour model. The MMP‐9, MMP‐2, α‐SMA, fibronectin, p‐PI3K, Smad2, Smad3, p‐Akt, p‐Smad2, p‐Smad3, Snail1, vimentin and Slug expressions were also decreased by resveratrol, and E‐cadherin in MDA‐MB‐231 cells was increased.^198^ All these results support the hypothesis that resveratrol in combination with nutlin‐3 would be able to induce programmed cell death at about half of the typical concentration of nutlin‐3 for the induction of apoptosis.^199^ A combination of resveratrol and cisplatin synergistically inhibited the MDA‐MB‐231 breast cancer cell viability and also inhibited TGF‐β1‐induced migration and invasion of MDA‐MB‐231cells by inhibiting EMT. Resveratrol was also reported to enhance antitumour effects and decrease adverse effects of cisplatin in MDA‐MB‐231 xenografts and involved in the mechanism for the effect of the regulations of the JNK, PI3K/Akt, NF‐κB and ERK expressions.^200^ Another study showed that the combination of sitagliptin (an antidiabetic drug) and resveratrol synergistically ameliorated clear cell renal cell carcinoma, and this effect was mediated by the decrease in TGF‐β1 and other inflammatory cytokines, such as TNF‐α, IL‐6 and STAT3.^201^ In irradiated mice, HS1793 (a resveratrol analogue) reduced tumour development by activating effector T cells. HS1793 treatment improved the outcome of radiation therapy by enhancing antitumour immunity. Additionally, HS1793 reduced the number of Tregs and lowered IL‐10 and TGF‐β secretion in irradiation tumour‐bearing mice.^202^

#### 6.2.13 Sulforaphane

Sulforaphane (Figure 3) is an isothiocyanate isolated from different vegetables of the crucifereae family, prominently from broccoli. Both preclinical and clinical studies underscore the ability of sulforaphane in preventing the development and/or suppressing the progression of various cancers.^203^, ^204^, ^205^, ^206^ The compound was studied against HepG2 hepatocellular carcinoma cells and also through a xenograft tumour growth model.^207^ This study revealed that sulforaphane has emerged as a safe compound for the treatment of hepatocellular carcinoma. The same study also confirmed that sulforaphane has the potential to inhibit TGF‐β‐induced EMT through the ROS‐dependent pathway.^207^

A recent study by Luo et al.^208^ was conducted to record the impact of sulforaphane on long non‐coding RNAs in pancreatic ductal adenocarcinoma using several human immortalised pancreatic duct cell lines. This study revealed that sulforaphane exhibited potential activity against pancreatic cancer, which was due to APOBEC3G downregulation that prevented Smad2 phosphorylation, and hence TGF‐driven pancreatic ductal adenocarcinoma progression.

#### 6.2.14 Thymoquinone

Thymoquinone (2‐isopropyl‐5‐methylbenzo‐1,4‐quinone, Figure 3) is mostly found as the biologically active compound in the oils of *Nigella sativa* seeds. Thymoquinone is considered a potential anticancer agent,^209^ and it plays dual roles, depending upon the molecular environment; it can act as an antioxidant or pro‐oxidant (by generating ROS in tumour cells).^210^ Thymoquinone inhibited the growth of cancer cells by inducing apoptosis, regulating the cell cycle and inhibiting the proliferation, migration and metastasis of tumour cells.^211^, ^212^ Its anticancer effect has been observed in various cancer types, such as lung, colorectal, prostate and breast cancer. In a recent study, thymoquinone was investigated as a radiosensitiser in the MCF‐7 and MDA‐MB‐231 breast cancer cells. Ionising radiation is considered to induce metastasis in cancer cells by eliciting TGF‐β, which is also known to regulate radio‐resistance. Thymoquinone downregulated E‐cadherin and cytokeratin 19, while mesenchymal markers, such as integrin aV, MMP‐9 and MMP‐2 were upregulated by irradiation treatment.^213^ Interestingly, in an in vivo study, decreased hepatic TGF‐β1 mRNA levels by 1.8‐fold were recorded from thymoquinone in hepatocellular carcinoma.^214^ Another in vivo study on renal cell carcinoma showed that TGF‐β1 might promote the metastasis in renal cell carcinoma, and thymoquinone has the ability to inhibit TGF‐β1‐induced metastasis.^215^

#### 6.2.15 Triptolide

Triptolide (Figure 3), a diterpenoid triepoxide compound, is extracted from different herbs used in Chinese medicine. Due to its considerable therapeutic effects, it has attracted considerable attention from researchers. It has been found to possess various pharmacological effects, such as antirheumatic, antimicrobial, anti‐inflammatory, immunomodulatory and antitumour activities.^216^, ^217^ Triptolide can produce cancer cell death by modulating apoptotic and autophagic pathways as well as inhibition of EMT and metastasis.^217^, ^218^

Triptolide inhibited the growth, migration and invasion of HT29 and HCT116 colon cancer cells with simultaneous reduction in the expression of VEGF and cyclooxygenase‐2. It also inhibited the expression of different TGF‐β receptor subunits (TGFΒRI and TGFΒRII).^219^ In another study, triptolide was tested against HT‐29 and SW480 colon cancer cells, where it reduced cell viability, metastatic potential and enhanced the apoptosis rate with simultaneous caspase‐3 and caspase‐9. Furthermore, it neutralised the changes caused by TGF‐β, such as alterations in E‐cadherin, N‐cadherin, vimentin and snail.^220^ In vivo studies showed that triptolide was able to decrease TGF‐ β, IL10 and VEGF in B16‐F10 xenograft mouse tumour model.^221^

#### 6.2.16 Withaferin A

*Withania somnifera*, commonly known as ashwagandha, or Indian ginseng, belongs to the family Solanaceae. its main constituents are alkaloids, steroidal lactone (withanolides and withaferins) and saponins.^222^ Withaferin A (Figure 3) isolated from *W. somnifera* was found to exert antineoplastic effects againg various in vitro and in vivo tumour models.^223^ This phytochemical was evaluated against MCF‐10A breast cancer cells and reported to partially reverse TGF‐β levels. During invasive breast cancer, EMT is usually characterised by low E‐cadherin and occludin levels and increased levels of the mesenchymal marker protein vimentin and fibronectin. However, after the treatment of withaferin A on MCF‐7 cells, there was an increase in the level of E‐cadherin and occludin, as well as suppression in vimentin. Withaferin A also led to the apoptosis of MDA‐MB‐231 and MCF‐7 by the intrinsic caspases pathway, without affecting vimentin levels.^224^

Withaferin A also inhibited the invasive property of human cervical cancer Caski cells and resulted in a reduction of MMP‐9 expression via Akt signalling pathway suppression. It was also found to inhibit TGF‐β‐induced Akt phosphorylation and caused downregulation of MMP‐9. This effect strongly supports the ability of withaferin A to control the invasiveness of the tumours.^225^

#### 6.2.17 Miscellaneous natural products and their synergistic effects

Polyphyllin, an active phytoconstituent of *Paris polyphylla*, exhibited a decrease in the tumour growth in the xenograft tumour model by antagonising facilitative effects of TGF‐β1.^226^ Similarly, andrographolide from *Andrographis paniculata* was also reported to decrease tumour growth and was bound to TGF‐β in an in vivo study for prostate cancer.^227^ The root extract of *W. somnifera* was studied on MDA‐MB‐231, MCF‐7 and T47D breast cancer cell lines and demonstrated a potential association between vimentin expression and cytotoxicity. Vimentin plays an important role in EMT's being an intermediate filament protein to promote metastasis. After treatment with the root extract of *W. somnifera*, TGF‐β did not inhibit expression of vimentin at the mRNA level but did on the protein level by disrupting its morphology in cells, finally inhibiting EMT.^228^

Marine natural products offer huge diversity but have limited explored resources. Marine products are enormous potential source reservoirs to isolate novel bioactive compounds from, and they also possess diverse chemical structures, which are potential sources for the drug discovery process.^229^ Marine organisms are valuable sources for novel anticancer agents and can be of prominent importance in the process of drug discovery.^230^, ^231^, ^232^, ^233^, ^234^, ^235^ Out of many marine products, very few have been investigated specifically for their effect on TGF‐β in cancer. For example, MS80 is a sulfated oligosaccharide (8000 Da) isolated from seaweed. This compound was reported to exhibit a potential inhibition of TGF‐β/Smad signalling in breast and lung cancer cell lines, and the compound also inhibited lung metastasis in orthotopic 4T1 xenografts by inhibiting TGF‐β.^236^

Natural products are very effective when utilised in association with other natural or synthetic agents. Studies have reported that the combined effect of natural products might improvise chemotherapeutic treatment effects against cancer cell proliferation.^237^ In a study on breast cancer, a combination of curcumin, endoxifen (a drug under development for breast cancer) and β‐estradiol (estrogen steroid hormone) was tested on MCF‐7 cell lines. The endoxifen upregulated EMT markers and altered MCF‐7 breast cancer cells’ morphology. However, curcumin addition did not prevent EMT activation. Endoxifen activated EMT and increased mRNA expressions of TGF‐β1; however, curcumin decreased TGF‐β1 mRNA expressions in comparison to endoxifen.^238^

Emodin, along with curcumin, was evaluated in cervical cancer cell lines SiHa and HeLa, which induced the EMT by the TGF‐β signalling pathway. The combination effectively downregulated the TGF‐β signalling pathway; hence, there was a decrease in the TGF‐β receptor II, P‐Smad3 and Smad4 expression level. Moreover, it also maintained the tumourigenic effects by inhibiting migration and invasion of cancer cells. Therefore, this combination had a synergistic chemotherapeutic effect against cervical cancer cells.^146^

## 7 TGF‐β‐ASSOCIATED AND ‐DEPENDENT OMICS APPROACHES IN CANCER THERAPEUTICS

To understand and subtype Wnt signalling‐driven malignancies and subsequent determination of relevant Wnt signalling‐targeted treatments, transcriptomic, genome sequencing and/or immunohistochemistry studies are of tremendous importance. These studies are also beneficial for the identification of the subset of patients that can get benefitted from the combination therapies with various signalling inhibitors.^239^ A multiomics data‐based study was performed to understand the molecular signatures linked with TGF‐β in gastrointestinal adenocarcinoma and a comprehensive neural network was framed. The results of this study suggested that TGF‐β can be a potential and possible drug target in gastrointestinal adenocarcinoma.^240^

**FIGURE 4:**
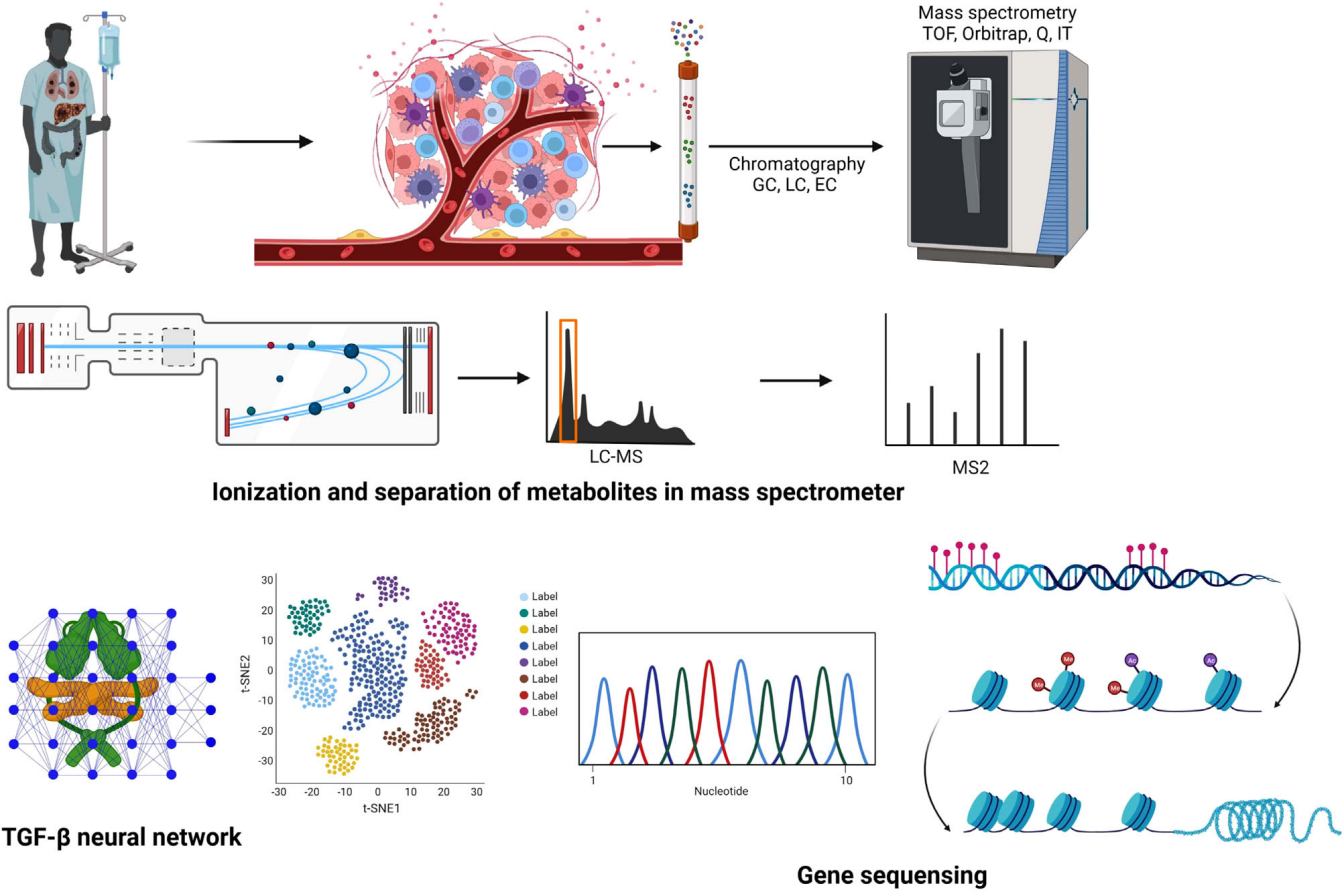
Schematic representation of various multiomics approaches for the mechanistic investigations of cancer. The image depicts omics‐based mechanistic evaluation of a multiple cancer patient's tumour environment with the help of metabolomics and genomics investigations. The image symbolically represents the utilisation of multiomics approach for the development of TGF‐β neural network

An explorative pathway analysis study conducted by Helleman et al.^241^ of various gene sets found that TGF‐β was a prominent gene associated with ovarian cancers that were chemotherapy‐resistant. Extra cellular matrix (ECM)/integrin‐mediated pathway induced by TGF‐β was considered as a potential target for ovarian carcinomas. This also showed that TGF‐β could play an important role in chemotherapy‐resistant cancers, and natural products targeting TGF‐β might show significant potential for such neoplasms. Various other proteomic and genomic analyses were conducted to explore the mechanism of TGF‐β in cancer progression.^242^, ^243^ However, a substantial gap is there, and more exhaustive and exclusive omics studies are needed to unravel the precise role of TGF‐β in tumourigenesis. A schematic representation of TGF‐β‐associated multidimensional omics approaches in cancer medicine is presented in Figure 4.

## 8 CONCLUSION, CURRENT LIMITATION, CHALLENGES AND FUTURE RESEARCH DIRECTIONS

Cancer is one the deadliest diseases known to man, the rate of which is increasing unexpectedly due to urbanisation, environmental issues and collective lifestyle changes. Although there are numerous significant medical and technological developments to combat this disease, many conventional cancer‐targeted therapies have severe side effects and complications, including exposure to severe toxicities and the development of resistance to the medications. Although cancer drugs act through various mechanisms involving different signalling pathways to treat individuals, we have narrowed the scope of our research to the TGF‐β signalling pathway. The TGF‐β signalling pathway plays a critical role in various cellular responses related to cell growth, apoptosis, motility, invasion, angiogenesis and differentiation. Although TGF‐β’s role is extensively studied for its tumour suppressive character during the early stages of cancer, it is also known to be involved in cancer progression, especially within the later stages. The possession of this unique property allows the pathway to act as a double‐edged sword, as it can play the roles of both tumour suppressor and promoter at different stages of cancer.

TGF‐β induces EMT in various epithelial cells, which is able to induce a stem cell‐like phenotype in cancer cells. Inhibiting TGF‐β signalling decreases the expression of protein markers and induces cell differentiation to less aggressive phenotypes. Clinical studies have revealed that drugs, such as galunisertib, show severe side effects in different phases of clinical trials. Several of these prominent side effects include a significant decline in lymphocytes, platelet count and white blood cells along with more common side effects, such as fatigue, nausea, constipation and alopecia.^244^, ^245^ Similar adverse events were also recorded for other drugs, such as fresolimumab.^246^ Due to the adverse effects pertaining to many of the TGF‐β inhibitors, usage of natural products may arise to become a better therapeutic approach for treating cancer due to their lesser amount of side effects in comparison to chemotherapy. In addition, natural compounds, such as curcumin, resveratrol, podophyllotoxin and emodin, can also be used as a bioenhancer for their synergistic effects to promote the anticancer potential. However, substantial clinical studies are required to have a proper understanding of the therapeutic efficacy of various natural products.

The prospective of natural agents in the field of drug discovery is boundless. A minimum of about one‐third of apex 20 drugs on the market are derived from natural products (specifically from plants), and approximately 50% of today's available and marketed drugs are categorised as naturally derived or designed based on natural products.^247^, ^248^

Therefore, treating cancer through targeting the TGF‐β pathway with various phytoconstituents is a new strategy that is most likely to result in fewer side effects for cancer patients. Despite ongoing research on natural product‐based cancer therapeutics many questions may arise, such as: (i) Do these natural products and their derivatives only kill cancer cells? If there is an effect on other types of cells, do they produce a favourable or harmful effect? (ii) Do these natural compounds and their derivatives have a synergistic effect with the conventional form of cancer treatment, or does their interaction induce unexpected effects? (iii) Does the use of natural products have advantages over the usage of conventional cancer treatment? Our review addresses these questions and provides comprehensive details about natural products and their derivatives, and whether they manage or inhibit cell proliferation with negligible effects on normal cells. To answer the second question, it is clear from multiple studies that natural products and their derivatives have highly synergistic effects and can be used as a bioenhancer to augment the anticancer effects of other chemotherapeutic drugs. Finally, additional evidence is available that shows the beneficial effects of natural products over conventional chemotherapeutic agents, specifically in terms of severe adverse effects of chemotherapy. However, comprehensive clinical studies are required not only to establish the anticancer effects of the natural products and their impact on TGF‐ β but also to evaluate the synergistic anticancer effects with other natural products and synthetic agents.

**FIGURE 5:**
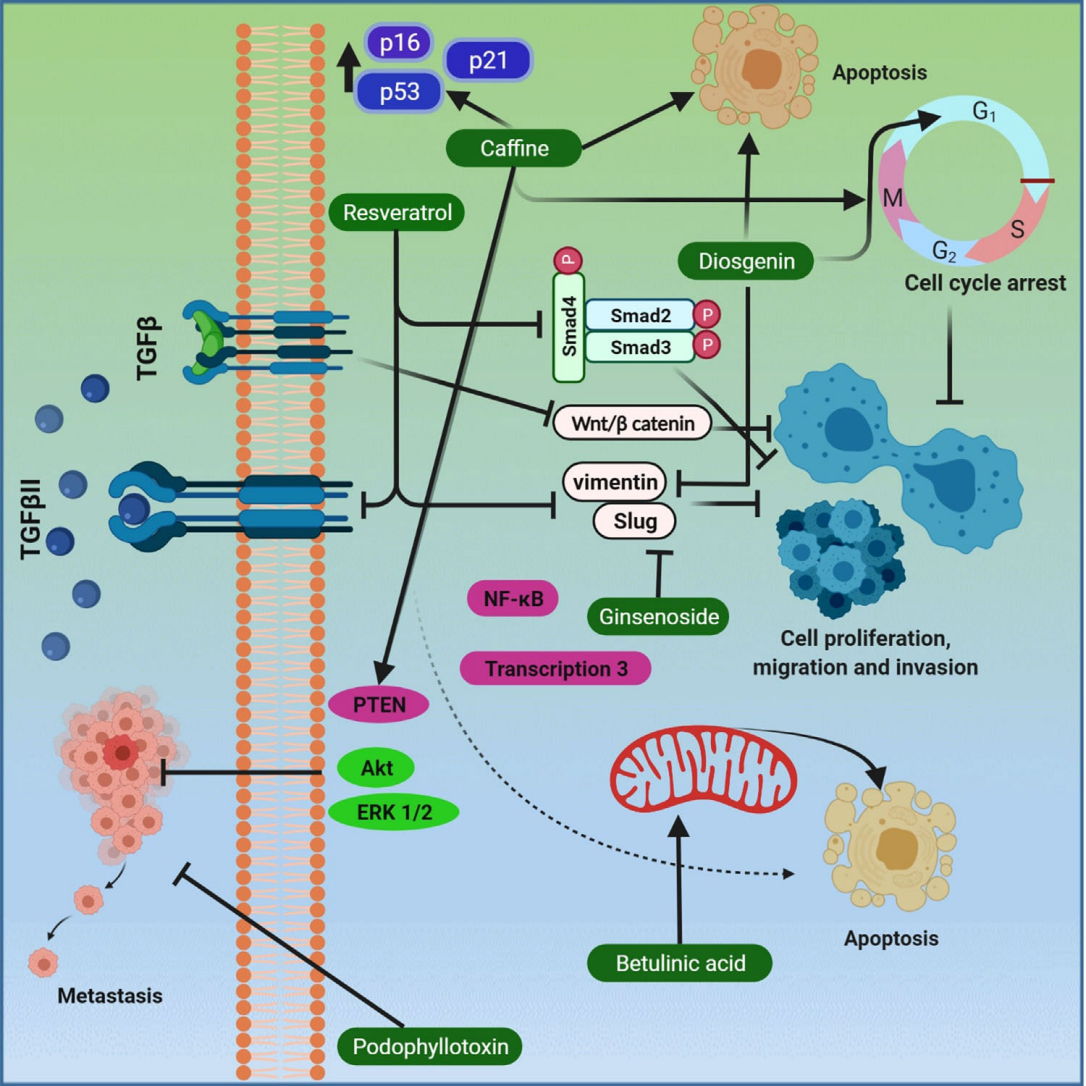
Mechanism of different natural compounds targeting the TGF‐β pathway in cancer. The figure illustrates the effects of various bioactive phytocompounds, such as betulinic acid, caffeine, diosgenin, ginsenoside, podophyllotoxin and resveratrol, on apoptosis, cell cycle, cell proliferation and metastasis linked to interference with the TGF‐β signalling

As demonstrated, cancer treatment with the usage of natural products has a long way to go within the area of preclinical and clinical studies. It is of utmost importance to optimise doses of natural compounds that will not lead to toxicity, but exhibit favourable pharmacokinetics and pharmacodynamics parameters, along with the precise mechanisms of action. As summarised in Figure 5, several bioactive natural compounds act through diverse mechanisms of the TGF‐βsignalling pathway. These include suppression of cell proliferation and inhibition of invasion and metastasis. Various natural compounds either directly inhibit TGF‐β signalling or can possibly act through mitochondria and induce apoptosis or cause cell cycle arrest at different stages.

Various natural products play important roles in the alteration of DNA methylation. Since there is a close association between the profiles of DNA methylation and TGF‐β signalling, a comprehensive investigation to better understand the value of TGF‐β as a target for anticancer natural agents is crucial. Several natural products have the potential of inducing TGF‐β production in the target cells, which might be valuable for cancer prevention. However, it could also be potentially harmful to patients with advanced‐stage cancer. Very few clinical studies pertaining to the effects of various natural products on TGF‐β signalling have been carried out; therefore, additional clinical studies are urgently warranted. We also recommend future studies utilising advanced pharmacological approaches, such as multiomics investigations. There is also a tremendous scope in the understanding of the involvement of TGF‐β at genomic levels. Additionally, the impact of various plant secondary metabolites can also be thoroughly investigated by using approaches, such as metabolomics. Based on emerging evidence as presented in this work, TGF‐β‐targeting bioactive compounds from natural sources can serve as potential therapeutic agents for the prevention and treatment of various human malignancies.
